# Design of a Base Station for MEMS CCR Localization in an Optical Sensor Network

**DOI:** 10.3390/s140508313

**Published:** 2014-05-08

**Authors:** Chan Gook Park, Hyun Cheol Jeon, Hyoun Jin Kim, Jae Yoon Kim

**Affiliations:** 1 Department of Mechanical and Aerospace Engineering/IAAT, Seoul National University, Seoul 151-741, Korea; 2 Department of Mechanical and Aerospace Engineering/ASRI, Seoul National University, Seoul 151-741, Korea; E-Mail: smartjhcz@snu.ac.kr; 3 Department of Mechanical and Aerospace Engineering, Seoul National University, Seoul 151-741, Korea; E-Mail: hjinkim@snu.ac.kr; 4 Hyundai Mobis, 17-2, Mabuk-dong, Giheung-gu, Yongin-si, Gyeonggi-do, 446-505, Korea; E-Mail: goairkim@gmail.com

**Keywords:** CCR, localization, sensor network, sensor node, TDC, TOF

## Abstract

This paper introduces a design and implementation of a base station, capable of positioning sensor nodes using an optical scheme. The base station consists of a pulse laser module, optical detectors and beam splitter, which are mounted on a rotation-stage, and a Time to Digital Converter (TDC). The optical pulse signal transmitted to the sensor node with a Corner Cube Retro-reflector (CCR) is reflected to the base station, and the Time of Flight (ToF) data can be obtained from the two detectors. With the angle and flight time data, the position of the sensor node can be calculated. The performance of the system is evaluated by using a commercial CCR. The sensor nodes are placed at different angles from the base station and scanned using the laser. We analyze the node position error caused by the rotation and propose error compensation methods, namely the outlier sample exception and decreasing the confidence factor steadily using the recursive least square (RLS) methods. Based on the commercial CCR results, the MEMS CCR is also tested to demonstrate the compatibility between the base station and the proposed methods. The result shows that the localization performance of the system can be enhanced with the proposed compensation method using the MEMS CCR.

## 1. Introduction

Wireless sensor networks consist of devices for monitoring physical world. In the sensor network, the field information detected by sensor nodes, for example convoy and routing of an enemy, is delivered to a user through a base station. To make this information meaningful, a sensor node localization technique is needed. This information should be provided with the information of the events to have validity. Localization methods in the wireless sensor network for Unattended Ground Sensors (UGS) have been researched for quite some time based on the Ubiquitous Sensor Network (USN) technology. Especially, the UGS system has been researched mainly in the U.S. and E.U. countries. In the U.S., Mini Intrusion Detection System (MIDS) was developed by Sandia Lab [1] and the Tactical Remote Sensor System (TRSS) is operated by the U.S. Navy. The Improved Remotely Monitored Battlefield Sensor System (IREMBASS) [2] of L-3 Communication Systems and localizing shooter by acoustic scheme [3] are all UGS systems for military operations. In the commercial sector, UC Berkeley has developed various sensor nodes such as Motes, MICA, PicoNode and Smart Dust and UCLA developed iBadge and Medusa MK-II [4]. MIT also has researched applications based on micro-sensor networks with the uAMPs [5]. In the E.U. countries, the Covert Local Area Sensor System for Intruder Classification (CLASSIC) system of Thales Defense Communications is being operated in the 12 NATO countries and 35 other countries [6]. Instead of the conventional RF sensor network system, there have been some trials for developing wireless sensor network systems based on an optical communication scheme. The wireless optical signal provides great security due to its directionality and also provides the surroundings for passive communication, which is efficient for saving energy. Nevertheless, research related to optical wireless sensor network systems has been done within a limited area such as sensor node development and optical communication. For example, the Smart Dust project of UC Berkeley and DARPA developed micro-scale sensor nodes with CCR, capable of passive communication using optical signals [7,8]. Some researches localize target objects using lasers for the purpose of preventing satellites from colliding with debris [9,10]. As mentioned earlier, the sensor nodes can sense many things. For example, sensor nodes sense various pieces of information such as the path of movement of an enemy and also can guide a missile to its target position. In order to make these functions work properly, accurate sensor node positions should be known. However topics related to sensor node position estimation in optical wireless sensor networks have not been researched properly yet. This paper is dedicated to introducing a 2D position estimation scheme for sensor nodes in an optical sensor network and an implementation of a base station system for MEMS CCR. The CCR is the optical device which makes the reflected light maintain a the parallel direction with the transmitted light. As a follow-up study of the MEMS CCR which was fabricated for optical communication applications [11], research on a base station system for position estimation using this MEMS CCR is carried out. The performance of the base station is firstly evaluated with a commercial CCR. Several methods to reduce the estimation error of the sensor node are introduced in this paper. Finally we demonstrate that the implemented base station and the error reduction methods are compatible with the MEMS CCR. By these processes, we provide a system for finding and communicating with MEMS-sized sensor nodes and propose error reduction methods for accurate sensor node localization determined experimentally. From now on the term, ‘CCR’ refers to ‘Commercial CCR’ in this paper. The position estimation using the MEMS CCR will be specifically mentioned in a later section.

The content is organized as follows: Section 2 presents the 2D localization scheme using the Time of Flight (ToF) and Angle of Arrival (AoA) methods for the sensor node in the optical wireless sensor network and also covers the actual implementation of the base station. In Section 3, the performance of the base station is evaluated in terms of sensor node position error. Also, the sources of position error in the implemented system are analyzed. Section 4 introduces the error reduction methods based on the analysis performed in the previous section. Section 5 introduces tests for the sensor nodes equipped with MEMS CCR. After each error reduction method is applied, the performance enhancements are compared altogether in Section 5 for a MEMS CCR test. Finally, conclusions will be drawn in Section 6.

## 2. Sensor Node Localization Method

### 2.1. Sensor Node Localization Concept

In this paper, the ToF and AoA methods are used to localize sensor nodes using a single base station. ToF is the most frequently used distance measurement method. When the speed of the wave is known, the distance can be calculated from the flight time difference between the receiver and transmitter. The desirable feature of the ToF measurement is that the base station and the sensor nodes do not require any synchronization of each system clock. ToF can be obtained using the following Equation (1):

(1) $$\frac{c\times (t_{2}-t_{1})}{2}=\text{Distance}$$

where *t*_1_ is the time when the pulse is transmitted, and *t*_2_ is the time when the reflected pulse is received. However, to localize the 2D position of the sensor node using only ToF measurement, more than three base stations are required [11,12].

AoA is the direction angle measurement method. The base station can measure the direction angle of a sensor node using the incoming angle of the signal. To localize the 2D position of the sensor node using only AoA measurements, two angle measurements are required for the sensor node. In the optical wireless sensor network, the sensor node direction angle can be obtained using the characteristics of the CCR, because light reflected in the CCR maintains a parallel direction with the transmitted light. Assuming that the sensor nodes are scattered randomly in a 2-D plane then we can consider that the base station and sensor nodes have same vertical position, in other word, there is no vertical position. Also by this assumption, the base station scans the 2-D plane as follows: first, the base station starts rotating to scan the plane by scanning angle. If there is a sensor node at a specific direction angle and there are no obstacles between the base station and the sensor node, an optical connection between the two is possible. Then we can obtain the direction angle and the distance between the two at this moment. Therefore, we integrate the ToF and AoA methods and apply them to the base station for localizing sensor node position.

### 2.2. Conceptual Design of the Base Station

**Figure 1.**
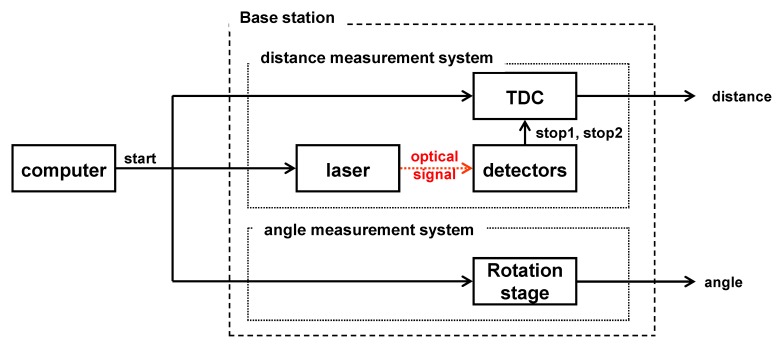
Block diagram of the ToF measurement system.

In this section, we design the base station system based on the methods previously mentioned in Section 2.1. For measuring the distance between the sensor node and the base station, a system as shown in Figure 1 was designed. The ToF measurement system mainly consists of the laser module which generates the laser pulses, the beam splitter, two detectors, and TDC module which can calculate the roundtrip time of the pulse, and all the system was controlled by a PC.

In order to measure the direction angle between the sensor node and the base station using the AoA, an encoder which can measure the rotation angle of the base station is used. As long as the sensor nodes are scattered on the plane with different direction angles and the base station has an optical connection with the sensor nodes, the base station can measure the direction angle of the sensor nodes during the rotation.

**Figure 2.**
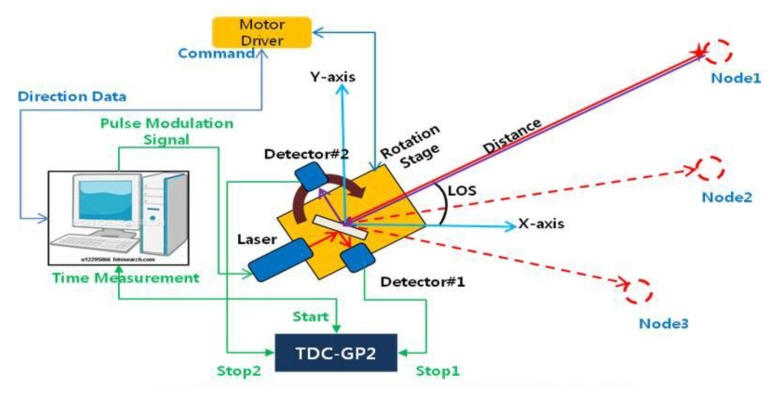
Design of the base station system.

The distance and direction angle data should be acquired at the single base station, and we designed the base station to be capable of measuring ToF and AoA together. The designed base station can seek the sensor nodes placed at the specific direction angles where the optical connection is possible, and can measure the distance between the detected nodes and the base station using optical pulses. Figure 2 shows the final design of the base station. With this scheme, the base station can localize the 2D position of the sensor nodes.

**Figure 3.**
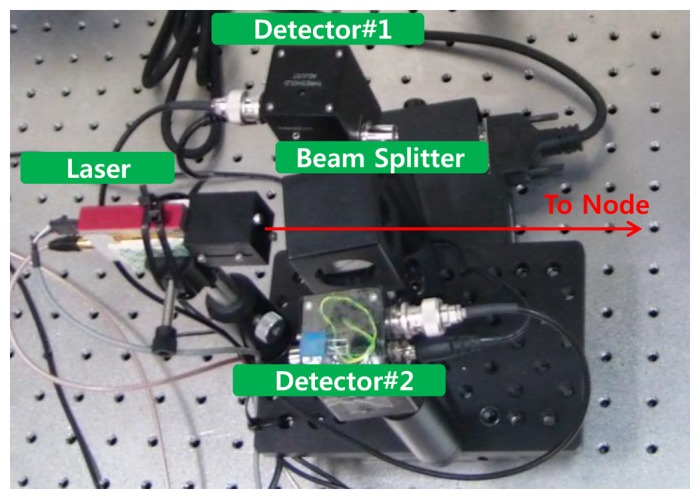
Implementation of the base station.

Based on the design of the previous section, we implemented the base station which can detect sensor nodes using optical signals. For security reasons, a near infra-red laser (wavelength = 905 nm) was used as the optical signal source. The pulse generated on the laser module is separated into two optical paths with the beam splitter. The pulse on the one of the paths is detected on the detector 1 and the pulse on the other path gets to the sensor node. The reflected optical pulse at the sensor node reaches inside again and is detected by the detector 2. The detected time difference between detector 1 and detector 2 can be used for measuring the distance. Also, the direction angle can be acquired when the detector 2 detects the reflected optical signal. Figure 3 shows the implemented system.

**Figure 4.**
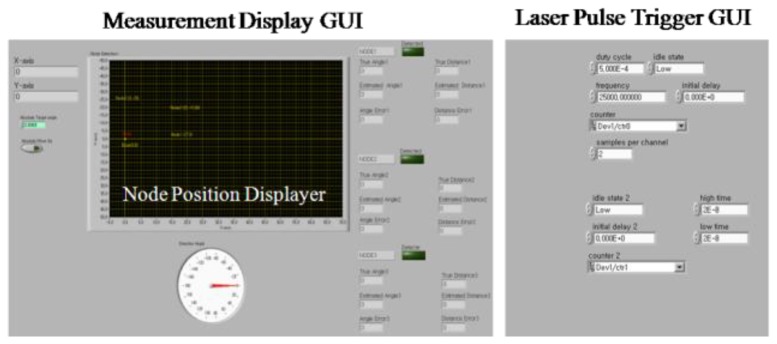
System GUI.

To control the base station, a Graphic User Interface (GUI) based on the LabView software was implemented. The implemented GUI displays the measured position of the sensor node and stores the measured data for post processing. Figure 4 shows the implemented GUI.

## 3. Performance Evaluation of the Base Station

### 3.1. Distance Measurment Evaluation Using ToF

**Figure 5.**
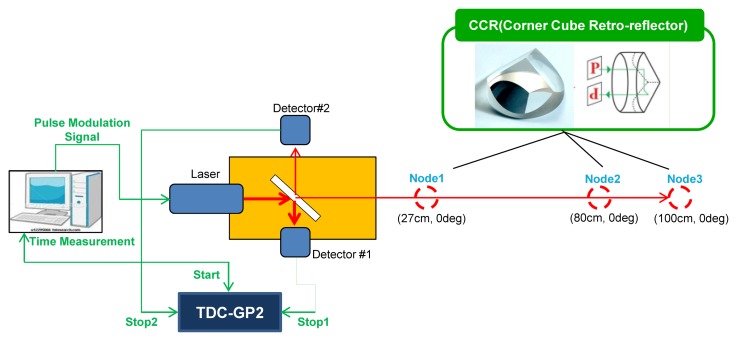
Node position for the evaluation of the distance measurement performance.

**Figure 6.**
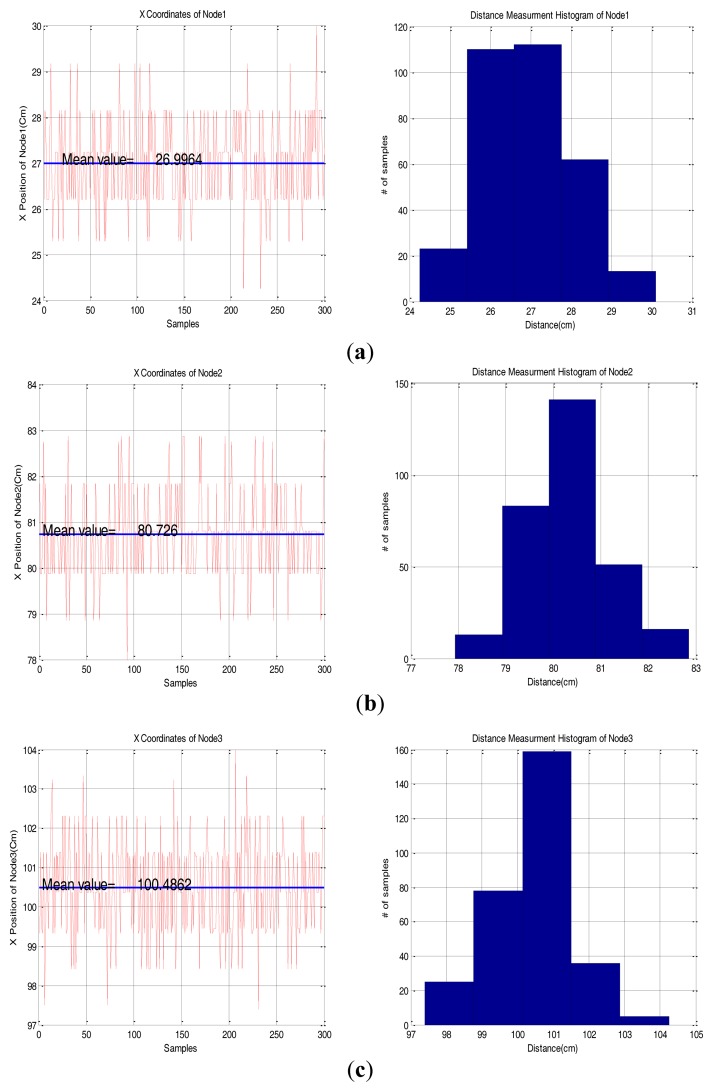
Measured distance data. (**a**) Node l: (27 cm, 0 deg), (**b**) Node 2: (80 cm, 0 deg), (**c**) Node 3: (100 cm, 0 deg).

A testbed was set up for the evaluation of the implemented base station. Figure 5 shows the testbed configuration. Table 1 shows specifications of the base station. Setting the base station at point (0,0), we placed three sensor nodes at (27 cm, 0 deg), (80 cm, 0 deg), (100 cm, 0 deg) and evaluated the measurement accuracy with the measured distance between these locations. Figure 6 shows the result of the position measurement. In this test a commercial CCR was used as the reflective material of the sensor node.

Figure 6 shows that values measured by the Time to Digital Converter (TDC) represent a discrete probability distribution because the TDC has 50 ps resolution rms, ±1.5 cm. Figure 6 also shows that the maximum distance error of each position is less than 4.5 cm and the histogram of each node shows that the samples which are close to the true distance have more chance to be measured. Table 2 also shows that the error of a 300-sample mean value is less than 2 cm for each distance test. Considering the sensor network environment, this precision is enough for the base station because the error value is within 10% of the measurement range.

### 3.2. Sensor Node Position Error Analysis

**Figure 7.**
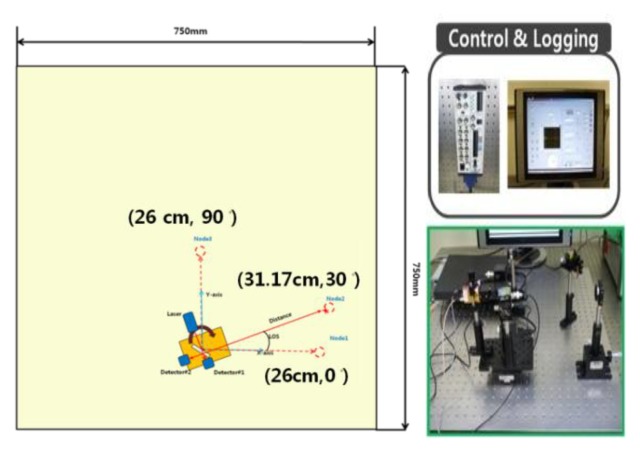
Testbed for the evaluation of the sensor node localization performance.

In this paper, scanning is defined as the rotation of the base station to search for the sensor node position. To evaluate the performance of the base station during the scanning, we placed several sensor nodes with different direction angles on the testbed and acquired the sample distribution of the base station measurements. The sensor nodes are placed at (26 cm, 0 deg), (31.17 cm, 30 deg), (26 cm, 90 deg) and (100 cm, 0 deg) as shown in Figure 7. Three hundred data samples are collected and the scanning resolution is set as 0.1 deg. These experimental settings are enough to show the sensor node measurement characteristics. We also set the scanning resolution at 0.1 deg and the clockwise rotation as the positive direction and the counter-clockwise one as negative in this test.

**Figure 8.**
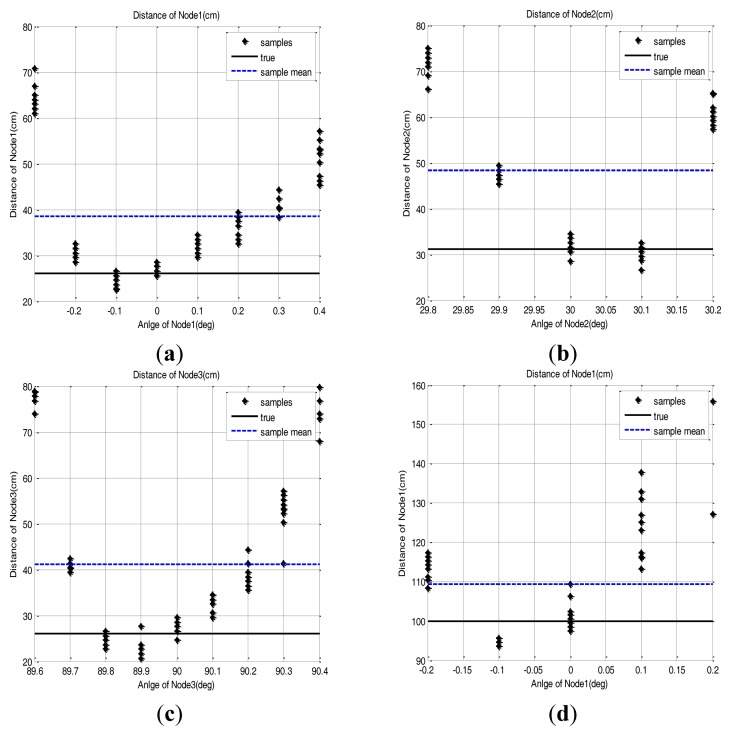
Scanning result with 0.1 deg resolution. (**a**) Node 1: (26 cm, 0 deg), (**b**) Node 2: (31.17 cm, 30 deg), (**c**) Node 3: (26 cm, 90 deg), (**d**) Node 1: (100 cm, 0 deg).

The measured samples are found to be distributed in the area near to the true position with a position error which changes with the direction angle. These results are shown in Figure 8. As the rotation angle of the base station goes farther from the true direction, the distance measurement is more distorted. This phenomenon is commonly observed in all the sensor nodes. Table 3 shows the position error value of the sensor node when a simple mean of sample positions is used for sensor node position estimation. We assume that the cause of this phenomenon is that if the direction angle is mismatched, then the reflective optical signal is only partially detected by the optical detector. Also, we assume that the partial detection causes the distortion of the pulse shape, which in turn affects the time measurement between the pulse edges.

**Figure 9.**
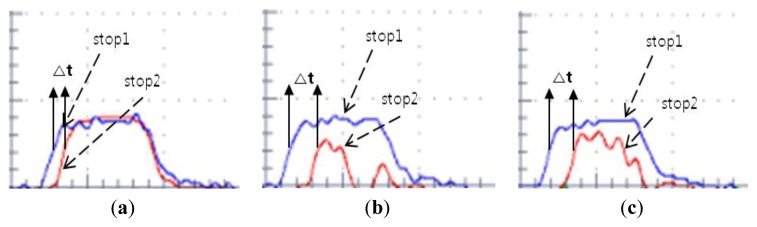
Detected pulse shape at Node 1 (26 cm, 0 deg). (**a**) 26.0492 cm at 0 deg (Normal case), (**b**) 49.3872 cm at 0.3 deg, (**c**) 64.6046 cm at 0.4 deg.

**Figure 10.**
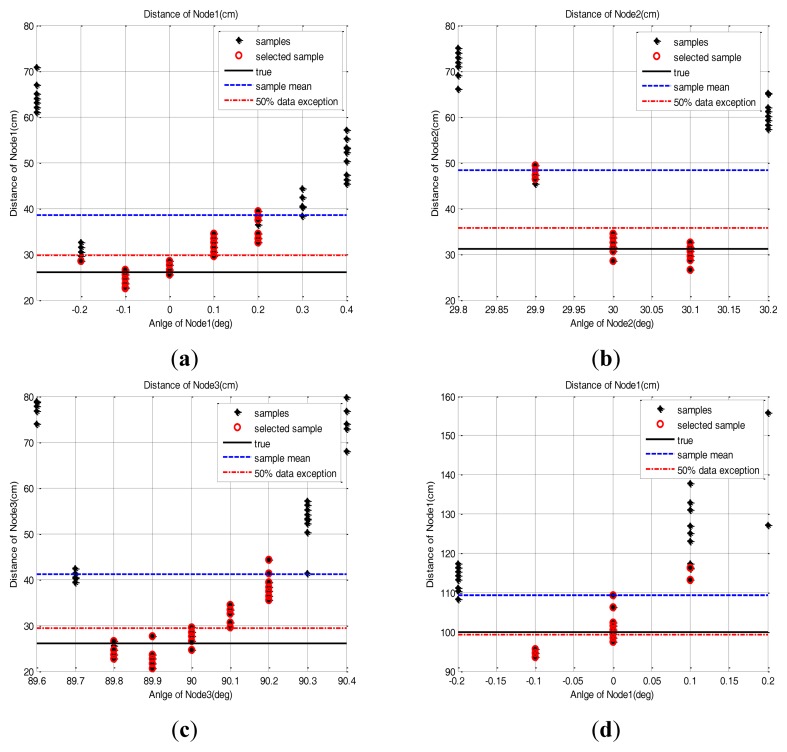
The performance comparison of the sensor node position estimation between the sample mean method and the 50% outlier exclusion method. (**a**) Node 1: (26 cm, 0 deg), (**b**) Node 2: (31.17 cm, 30 deg), (**c**) Node 3: (26 cm, 90 deg), (**d**) Node 1: (100 cm, 0 deg).

For validating the assumption, we scanned the Node 1 and observed the detected pulse shape by detector 2 with an oscilloscope. Figure 9a shows the pulse shape obtained when the base station points out the true angle, 0 deg. Figure 9b,c shows the pulse shapes when the base station points out angles that are different from the true direction. Figure 10b,c shows that the detected pulse shape at different angles is distorted and the voltage level is decreased compared to the pulse in the true direction angle shown in Figure 9a. Therefore, based on the tendency of the result, we found that the more the rotation angle of the base station is mismatched with the true direction angle, the larger the distortion and voltage level drop presented in the detected pulse. The distance measurement at each angle is also presented in Figure 9. These values show that as the rotation angle of the base station goes farther from the true direction angle, the distance measurement is more distorted.

In this test, we found that the angle mismatch contributes to the decrease of the detected pulse voltage level. Therefore we can conclude that as the rotation angle of the base station goes farther from the true direction angle of the sensor node, the intensity of the reflected pulse is decreased and this intensity drop causes the distortion of the distance measurement.

## 4. Sensor Node Position Error Reduction Techniques

There is no pre-acquired information about the sensor node position in real operational environments. Therefore the sensor node position should be estimated based only on the measurement of each sensor node. However, if the range and angle measurement error is included in the acquired samples, the error will have direct effects on the position estimation with the simple mean of the samples. To reduce such estimation error, several correction methods are introduced. In this section, to reduce the effect of the error, correction methods are applied to the acquired samples and the position estimation error of each method is evaluated. From the measured sample data of Figure 8, we observed that a higher position error was found at both edge sides of the sample group. The methods we used are data exclusion and RLS. Each method is explained in the following sections and the result of 20 repeated tests are covered in the following section.

### 4.1. 50% Outlier Exclusion of Outside Edges

To minimize the effect of the existing error, the samples near the outside edges are excluded using the 50% outlier exclusion of the outside edges as followings:

(2) $$\begin{array}{ll} {\hat{x}}_{i} & =\frac{\overset{\left\lfloor 0.75\times m_{i}\right\rfloor }{\underset{k=\left\lceil 0.25\times m_{i}\right\rceil +1}{\text{∑}}}r_{k}\cos\theta_{k}}{\left\lfloor 0.75\times m_{i}\right\rfloor -k+1}\\ {ŷ}_{i} & =\frac{\overset{\left\lfloor 0.75\times m_{i}\right\rfloor }{\underset{k=\left\lceil 0.25\times m_{i}\right\rceil +1}{\text{∑}}}r_{k}\sin\theta_{k}}{\left\lfloor 0.75\times m_{i}\right\rfloor -k+1} \end{array}$$

where subscript *i* means the *i*-th node, *m_i_* is the number of *i*-th node measurement. Also, *r_k_* is the distance, and *θ_k_* is the angle. The ⌊ ⌋ sign means that the largest integer less than or equal and the ⌈ ⌉ sign means the smallest integer greater than or equal.

As in the Equation (2), the sample group of one sensor node, of which edge samples on both sides are deleted in as many as 50% of the total samples, are averaged to estimate the 2D position of the sensor node. The distance estimation result based on this method is presented in Figure 10. Because of the parabolic measurement characteristics of sensor nodes, the samples on both sides have more errors. Therefore, the 50% outlier exclusion method has an effect eliminating the samples in order of larger error.

### 4.2. Weighted Recursive Least Square Method

**Figure 11.**
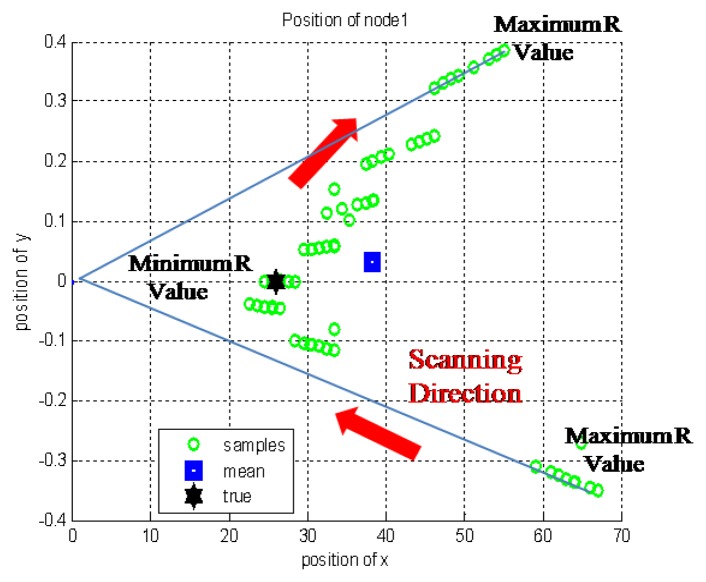
The concept of the weighted RLS method.

**Figure 12.**
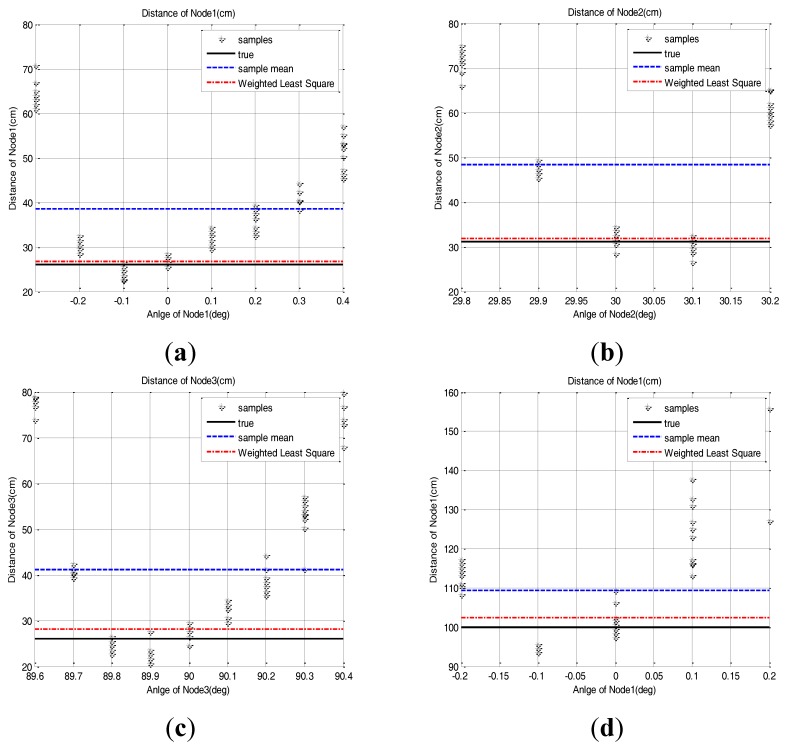
The performance comparison of the sensor node position estimation between the simple mean method and the weighted RLS method. (**a**) Node 1: (26 cm, 0 deg), (**b**) Node 2: (31.17 cm, 30 deg), (**c**) Node 3: (26 cm, 90 deg), (**d**) Node 1: (100 cm, 0 deg).

Using the characteristic that the higher position error was found at both the edge sides of the sample group, the weighted RLS method can be applied to the position estimation of the sensor node. This method uses the adaptation of the measurement noise covariance, R. This value R is increased as the samples approach the edges of the sample group of one node to reduce the effect of the error existing in the samples. This concept is well described in Figure 11 and the mathematical representation of the RLS scheme is given by:

(3) $$\begin{array}{c} {ẕ}_{k}={\underline{x}}_{k}+{\underline{v}}_{k}\\ K_{k}=P_{k-1}{H_{k}}^{T}{\left[H_{k}P_{k-1}{H_{k}}^{T}+R_{k}\right]}^{-1}\\ P_{k}=(I-K_{k}H_{k})P_{k-1}\\ {\hat{\underline{x}}}_{k}={\hat{\underline{x}}}_{k-1}+K_{k}[{ẕ}_{k}-H_{k}{\hat{\underline{x}}}_{k-1}] \end{array}$$

where the *z_k_* is the distance measurement, *x_k_* is the true distance, *v_k_* is the measurement distortion, *H_k_* is measurement matrix and *P_k_* is the error covariance matrix [13]. The distance estimation result based on this method is presented at the Figure 12. By scanning with a base station which rotates the scanning resolution respectively, the parabolic measurement characteristics of sensor nodes show that the number of measured direction angles is an odd or even number and the true direction angle is in the center of them. In this experiment, if the number of measured direction angles is odd, measured symmetrically, we assign 90% weighting to samples at the center angle and 10% weighting to the rest of the samples at the rest angles symmetrically. If the number of measured direction angles is even, we assign 45% weighting to the samples at the two center angles, respectively, and 10% weighting to the rest symmetrically. Because we allocate high confidence to the center angle, the resulting mean distance is affected by samples in the center angle and the mean error is reduced considerably.

### 4.3. Comparison of the Results

**Figure 13.**
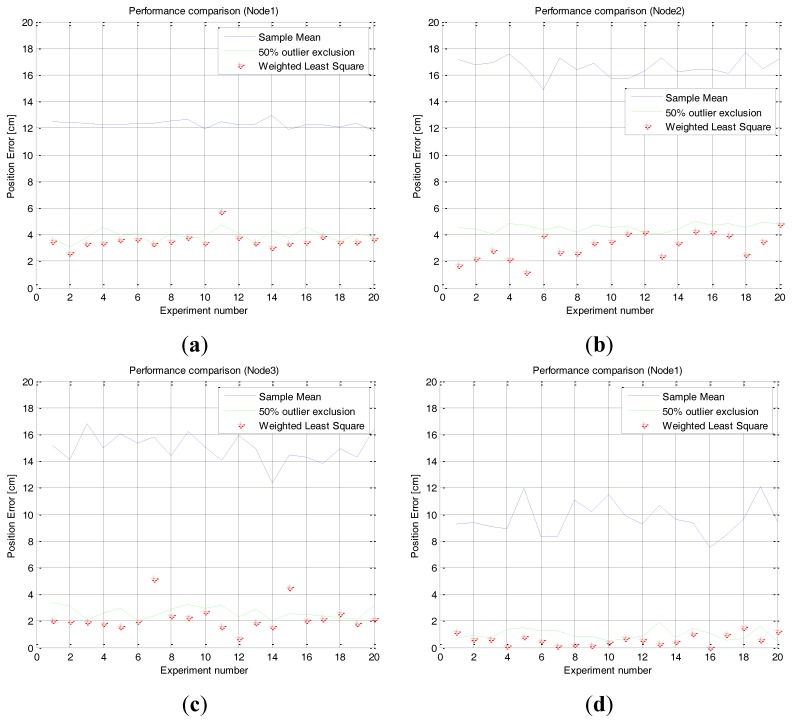
Position error of the repeated tests with the error reduction methods. (**a**) Node 1: (26 cm, 0 deg), (**b**) Node 2: (31.17 cm, 30 deg), (**c**) Node 3: (26 cm, 90 deg), (**d**) Node 1: (100 cm, 0 deg).

The scanning tests are repeated 20 times and each proposed method is applied. Figure 13 shows the estimated position error of the repeated tests when each method is applied. The average and standard deviation of the repeated tests are shown in Table 4.

The result shows that the method using 50% outlier exclusion of the outside edges and the weighted RLS method can effectively reduce the position error of the sensor nodes compared to the mean of raw samples. It also shows that the weighted RLS method shows a little bit more stable performance than the method using 50% outlier exclusion of the outside edges. Because of the symmetric and parabolic measurement characteristics of sensor nodes, the two methods have an effect that gives more confidence to samples near the center angle, but the weighted RLS has an effect that gives much more confidence to samples at the center angle than the 50% outlier exclusion method. Therefore the net result is that the weighted RLS method is a little bit better.

## 5. Base Station Performance Evaluation Using MEMS CCR

### 5.1. Compatibility Test for the MEMS CCR

**Figure 14.**
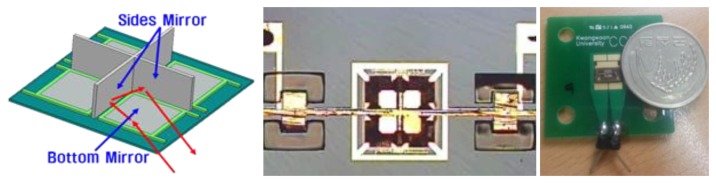
Tested MEMS CCR structure and design.

In this paper, a CCR fabricated with MEMS technology for optical communication applications is tested to show the compatibility of base station with micro-sized sensor nodes, which are compact and save energy. Such a MEMS CCR consists of three square mirrors of 300 μm and it functions the same as the commercial CCR [8]. The MEMS CCR used in this test is shown in Figure 14. MEMS CCR has a different structure and physical characteristics from the commercial CCR, and the compatibility of the implemented system and the validity of the proposed error reduction methods are demonstrated for the MEMS CCR. Therefore, in this test, we will show that the implemented base station can estimate the sensor node position and the estimated position error can be reduced with the proposed techniques.The true position of the MEMS CCR embedded sensor node is (26 cm, 0 deg) and the scanning resolution is 0.1 deg.

**Figure 15.**
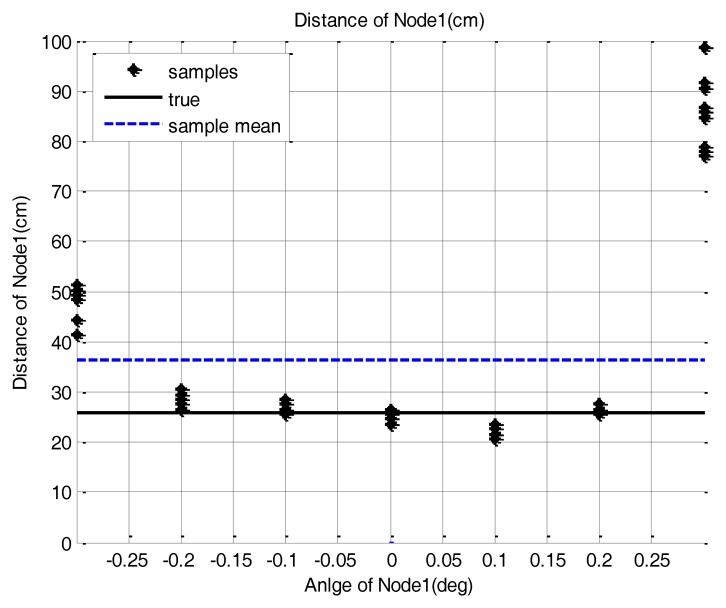
Scanning result of MEMS CCR with the 0.1 deg resolution.

With the scanning test, we observed that the MEMS CCR can be detected with the implemented base station. However, the sample distribution was different from that of the commercial CCR. Figure 15 indicates that the samples have more consistent distribution around the true position than the samples of commercial CCR do. Therefore in this section, we conclude that the implemented base station is compatible with the MEMS CCR.

### 5.2. Result of Error Reduction Techniques for the MEMS CCR

**Figure 16.**
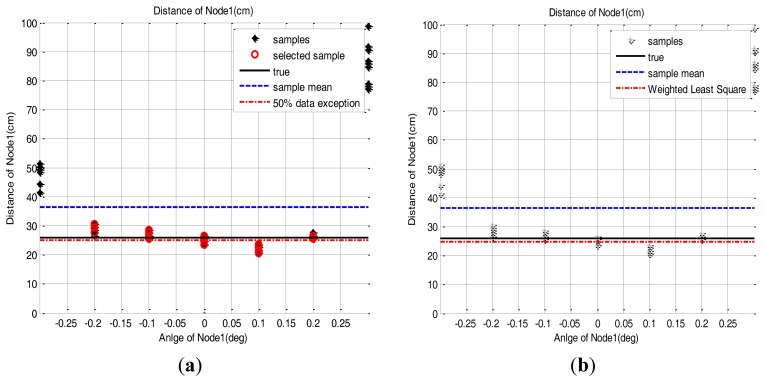
Position error of the repeated tests with the error reduction methods. (**a**) 50% data exclusion method, (**b**) RLS method.

In this section, the correction methods are applied to the data from the MEMS CCR. Like the tests in Section 4, the 50% data exclusion method and RLS method are applied to the samples of the MEMS CCR test. Figure 16 shows the result of each method.

**Figure 17.**
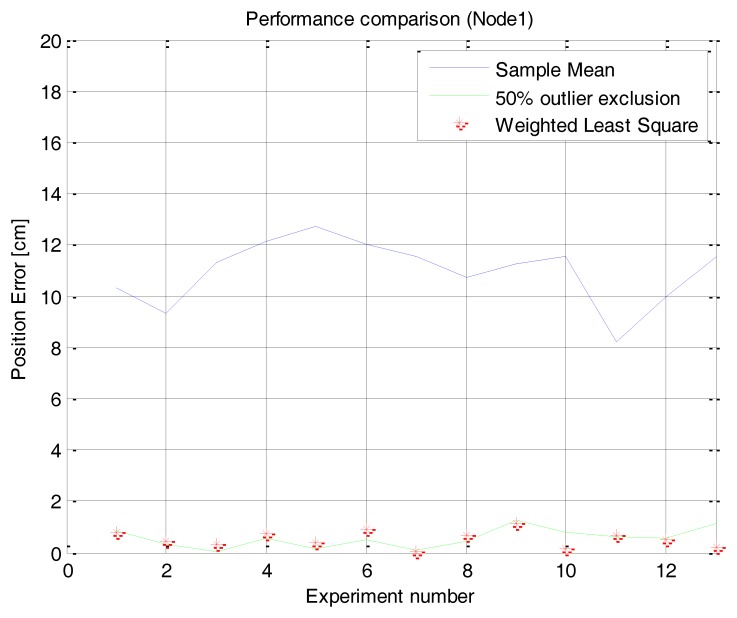
Position error of the repeated tests with the error reduction methods for the MEMS CCR.

We performed this test several times and applied both methods. Table 5 shows the mean and standard deviation of the position error data obtained through the repeated tests. Figure 17 shows the results of each test trial. Compared to the samples of Section 4, the inclination of the curve is a little bit changed but the parabolic shape still remains. Because of this, the weighted RLS method still shows less estimation error than the 50% outlier exclusion method, but the difference is just about 0.03 cm. From this experiment, we can conclude that the proposed correction methods can effectively reduce the position error of the MEMS CCR.

However, the performance difference between the two methods is less than 0.18 cm, which is just 0.7% of the true distance, 26 cm. Therefore, through these repeated tests using the MEMS CCR, both proposed methods can reduce the error of estimated position compared to the case using the simple sample mean method.

In Section 4, the number of samples decreases due to the declination of the field of view as the distance is expanded, but the sample distribution keeps its parabolic shape as the distance increases. Based on the results in Section 4, the methods are shown to be valid at the different distance. Therefore, we conclude that two proposed methods and the implemented base station are compatible with the MEMS CCR and can localize sensor nodes embedded with MEMS CCR.

## 6. Conclusions

We evaluated the sensor node localization performance by implementing a base station system. Through many experimental results, it is claimed that the implemented base station system is designed for a wide use and the accurate position of MEMS CCRs can be obtained. The two error reduction methods not only show the common feature that they decrease the effect of the distorted sample, but also differences in the way they decrease the effect. The outlier exclusion method simply excludes the outlier samples, but the weighted least square method steadily decreases the effect of erroneous samples. Finally we draw the conclusion that both the outlier exclusion method and weighted least square method can reduce the estimated position error compared to the case using the simple sample mean method.

## Figures and Tables

**Table 1.** Specifications of the system.

	**Item**	**Details**
**Base Station**	Pulsed Laser Diode Module	Wavelength: 905 nm, Power : 25 mW
	Precision Rotation Stage	Travel Range: 360°, Minimum Incremental Motion : 0.0015°
	Photodetector	Model: ET-2030TTL, Sensitivity : 0.4 A/W @ 830 nm
	Time to Digital Converter	Model: ATMD-GP2, 50 ps resolution rms, Measurement Range: 0 to 1.8 μs

**Sensor Node**	Commercial CCR	Model: N-BK7 Corner Cube Retroreflector, Inner diameter 7.16 mm

**Table 2.** Performance evaluation of the distance measurement system.

**Node#**	**Node 1**	**Node 2**	**Node 3**

**Data**			
True (cm)	27	80	100
Mean (cm)	26.9964	80.726	100.4862
Difference (cm)	0.0036	0.726	0.4862
Std. (cm)	0.9723	0.8946	1.2077

**Table 3.** Position difference between true value and estimation value using the simple mean.

**Node#**	**Data**
Node 1 (26 cm, 0 deg)	12.5086 cm
Node 2 (31.17 cm, 300 deg)	17.1501 cm
Node 3 (26 cm, 90 deg)	15.2036 cm
Node 1 (100 cm, 0 deg)	9.2401 cm

**Table 4.** The mean error and standard deviation of the repeated tests.

**Node#**	**Node 1 (26 cm, 0 deg)**		**Node 2 (31.17 cm, 30 deg)**		**Node 3 (26 cm, 90 deg)**		**Node 1 (100 cm, 0 deg)**	
	
**Methods**	**Mean Error (cm)**	**Std. (cm)**	**Mean Error (cm)**	**Std. (cm)**	**Mean Error (cm)**	**Std. (cm)**	**Mean Error (cm)**	**Std. (cm)**
Sample Mean	12.3136	0.2613	16.5935	0.6745	14.9827	1.0308	9.7024	1.1985
50% Outlier Exclusion	3.9201	0.4060	4.5437	0.2791	2.6414	0.4278	0.9935	0.3977
Weighted Least Square	3.5947	0.5651	3.2072	0.9509	2.2658	0.9529	0.6426	0.4048

**Table 5.** The mean error and standard deviation of the repeated tests for the MEMS CCR.

**Node#**	**Node 1 (26 cm, 0 deg)**	
	
**Methods**	**Mean Error (cm)**	**Std. (cm)**
Sample Mean	10.9774	1.1970
50% Outlier Exclusion	0.5623	0.3617
Weighted Least Square	0.5348	0.3090
